# Impact of CMR parameters on prognosis after ST-elevation myocardial infarction - a comparison to traditional outcome markers

**DOI:** 10.1186/1532-429X-14-S1-O4

**Published:** 2012-02-01

**Authors:** Suzanne de Waha, Ingo Eitel, Steffen Desch, Georg Fuernau, Philipp Lurz, Matthias Gutberlet, Gerhard Schuler, Holger Thiele

**Affiliations:** 1 Department of Internal Medicine/Cardiology, University of Leipzig, Heart Center, Leipzig, Germany; 2 Department of Diagnostic and Interventional Radiology, University of Leipzig, Heart Center, Leipzig, Germany

## Summary

In patients with STEMI, CMR parameters such as infarct size, MO and the MSI add incremental prognostic value above traditional outcome markers.

## Background

Infarct size, microvascular obstruction (MO) and the myocardial salvage index (MSI) assessed by cardiac magnetic resonance imaging (CMR) have been shown to be associated with adverse clinical outcome. However, in daily clinical routine prognosis after STEMI is evaluated using simple assessable parameters such as ST-segment resolution, enzymatic infarct size, pre- and post-PCI TIMI-flow, left ventricular ejection fraction (LV EF) and TIMI-risk score. Up to date it remains unclear if the assessment of infarct size, MO and MSI by CMR adds incremental prognostic value in comparison to the traditional outcome markers.

## Methods

STEMI patients reperfused by primary angioplasty (n=392) within 12 hours after symptom onset underwent CMR 3 days after the index event (interquartile range [IQR] 2-4). Infarct size and MO were measured 15 min after gadolinium injection. T2-weighted and contrast-enhanced CMR was then used to calculate MSI. In all patients ST-resolution, pre- and post-PCI TIMI-flow, as well as the enzymatic infarct size using creatine kinase-MB were recorded. All patients underwent transthoracic echocardiography for the assessment of LV EF. Finally the TIMI-risk score was calculated. Clinical follow-up was conducted after 19 months (IQR 10-27). The primary endpoint was defined as a composite of death, myocardial reinfarction and congestive heart failure. C-statistics were computed to assess the prognostic value of different models regarding the occurrence of the primary endpoint.

## Results

Comparison of a model including only traditional outcome markers (model 1: ST-resolution, enzymatic infarct size, pre- and post-PCI TIMI-flow, LV EF and TIMI-risk score) to a model including only CMR parameters (model 2: infarct size, MO and MSI) revealed an increase of the corresponding c-statistics (model 1: 0.68 vs. model 2: 0.73, p=0.03). Comparison of model 1 to a model including the CMR parameters on top of traditional outcome markers (model 3) demonstrated an incremental prognostic value of infarct size, MO and MSI (model 1: 0.68 vs. model 3: 0.77, p=0.02).

## Conclusions

In patients with STEMI, CMR parameters such as infarct size, MO and the MSI add incremental prognostic value above traditional outcome markers.

## Funding

None.

